# Protocol for a scoping review of the current data practices in forensic medicine

**DOI:** 10.1186/s13643-020-01308-7

**Published:** 2020-04-08

**Authors:** Salona Prahladh, Jacqueline van Wyk

**Affiliations:** 1 Department of Forensic Medicine, Inkosi Albert Luthuli Central Hospital, 800 Vusi Mzimela Rd, Umkumbaan, Durban, 4091 South Africa; 2 Discipline of Clinical and Professional Practice, College of Health Sciences, University of KwaZulu-Natal, Durban, South Africa

**Keywords:** Autopsy, Postmortem examination, Forensic pathology, Forensic medicine, Data, Database

## Abstract

**Background:**

Data related to forensic postmortems or autopsies are still mainly captured in hard copy format and archived. This paper-based practice impacts on the practitioner’s ability to report on incidence, prevalence, and statistical trends related to cases that are commonly seen in mortuaries in forensic medicine. An autopsy can be used to inform and provide evidence-based knowledge for further research about important issues, including social development and assist in providing statistics and data for public health initiatives for implementation and monitoring. Currently, in forensic medicine and pathology research developments are largely hampered by the inefficient data capturing system which only allows access to basic information while pertinent information is largely recorded manually and is therefore difficult to obtain. There is thus a need to improve the efficiency of the data capturing system in forensic pathology, and this review is intended to inform the choice and decisions of appropriate data capture practices and is being conducted to identify nationally and internationally the current data mining and storage systems in place.

**Methods:**

The methodology for this scoping review will be guided by the methodological framework for scoping review. The search strategy was developed by the authors, and we will conduct a search from 1 January 2008 of electronic databases (Cochrane Library, Scopus, Web of Science, and Science Direct) and search through WorldCat and PubMed for citations and literature using both keywords and the Medical Subject Headings (MeSH).The electronic search will be supplemented by hand searching references of the included studies and references in journals and websites. All articles will be assessed for eligibility by two reviewers (the primary and secondary authors) and uploaded into EndNote Excel spreadsheet, and duplicates will be identified and removed. The two reviewers (primary and secondary authors) will screen the eligible abstracts and articles against the inclusion criteria, and selection will be on a minimum percentage agreement of 50%. The selection process will be documented by following and using a PRISMA flow diagram. The extracted data will be analyzed and reported in the form of a narrative review with descriptive analysis and text analysis once the data is summarized for description and characterization.

**Discussion:**

The results of this review will identify and describe data capturing, management, and storage practices for use in forensic medicine. It will also review the efficiency of the different data systems and report where possible on the uses of the data system within the forensic medicine and pathology field.

**Ethics and dissemination:**

Although research ethics approval is not required for this scoping review because the study will not include human or animal participants, the study was submitted for approval to the University of Kwazulu Natal Biomedical Research Ethics Committee and obtained provisional approval. Data will be sourced only from published literature and gray literature. The results will be presented at relevant national and international conferences and published in a peer-reviewed journal. All search results including excluded studies will be added into an addendum in the article and made available for public perusal to therefore ensure transparency and reproducibility.

## Background

The discipline of forensic pathology involves the study of “unnatural” and sudden unexpected deaths. It is a medicolegal investigation process that forms part of the judiciary process, and therefore, consent from relatives is not necessary to perform the autopsy. The medicolegal autopsy is a legal requirement under the Inquests Act, Birth and Deaths Registration Act, Health Professions Act, and Criminal Procedures Act as per the Regulations of Forensic Pathology Services in South Africa [1, 2]. As part of the inquest procedure and the National Code of Guidelines for Forensic Pathology Services, an authorized medical doctor is appointed by the province to perform a postmortem. In South Africa, it is usually a medical professional trained in forensic medicine or a qualified forensic pathologist. But internationally, such as the USA, the coroner and medical examiner system are followed, and the jurisdiction differs in each state [3, 4]. The medical examiner is an appointed medical practitioner with board certification in medical specialty whereas a coroner is an elected person without professional training. A coroner may refer a case to a medical examiner if needed. Across the world, the death investigation systems may differ in accordance with law.

According to Statistics SA, the report on “Mortality and causes of death in South Africa, 2016: Findings from death notification”, the number of deaths were recorded at 456,612 (11%), and unnatural deaths were composed of approximately 51,242 (11%) deaths. One hundred fourteen thousand, four hundred eighty-nine (25%) postmortem examinations and 44,798 (10%) autopsies were performed in 2016 [5]. Internationally, although autopsies for external causes of death have increased to approximately 10%, autopsies for hospital-related causes of death declined by 58% [6, 7]. This large decline in hospital or clinical autopsies has been mainly attributed to religious reasons and technological advancements which presents a difficult but unique situation to the medical fraternity because autopsy pathology has provided great insight into disease processes and into disease surveillance and progression and therefore has played a large role in medicine to advancement of treatment [8–11].Recently, there has a resurgence in the twenty-first century to focus on patient safety and care, and quality assurance has recently been thrust into the spotlight of late due to patients being more informed and educated, human nature being generally inquisitive, and the widespread accessibility of information on the internet. Medical malpractice and misdiagnosis claims have seen a steady increase over the last decade [12]. Improving our current reporting system on medicolegal autopsies can be used to fill in the gaps in autopsy pathology, identify issues of high importance, and retrain medical personnel appropriately and will ultimately improve the quality of patient care which are important aspects of public health and health care in general. Postmortems provide important and underutilized information that are inaccessible if not captured appropriately. Internationally, it has been shown that secondary use of clinical data can be accelerated by modernizing our current health care infrastructure with regard to health records [13–16]. But currently, research is laborious and difficult, due to the unavailability of data which is only accessible presently manually or in hard copy format at each mortuary. The collection, storage, and dissemination of important health care information for use in academic departments and governmental organizations have advantages for public health such as epidemiology studies, health surveillance and to institute appropriate preventative measures [17–20]. Therefore, improving and streamlining data management in medicine are important to provide an intuitive system with appropriate tools to support new and ongoing projects [6]. While paper records are essential, the advancement of technology allows for data to be entered into a computerized database that can further be compared and analyzed, thereby greatly reducing laborious efforts of accessing paper-based information and increasing the utility of the information for teaching, research, epidemiology, and health monitoring. Medical data proves to be unique in a lot of aspects including heterogeneity, data types, confidential storage of privacy sensitive voluminous health records, and transmission of the information for ancillary use, and therefore, appropriate and adequate recording systems are a prerequisite [21–23]. There is a need to improve current data reporting systems in place to support forensic medicine as well as medicine [24, 25]. The objective of this scoping review is to gain insight on the different data capturing systems and how it has impacted on different departments in medicine with a special interest in forensic medicine and data capturing in autopsy reporting.

## Methodology

This scoping review will be conducted to gather evidence from the literature on the use, benefits, and application of electronic data information practices in forensic medicine and will be extended to similar academic health-related disciplines. The objectives are to identify and review current data practices for collection, storage, and management of health care records; examine the literature on the different methods used; explore and describe literature relating to the impact of current; and improve data collection and management practices in forensic pathology and medicine. It is anticipated that the results of this study will inform the medical fraternity, the public, and the government of the importance of good and efficient data capturing methods in health care advancement and inform on decisions relating to implementing such systems in the forensic medicine department.

## Scoping review

We will conduct a systematic scoping review of literature reporting on at least the last 10 years in data capturing practices and database management in forensic medicine and pathology locally and internationally and in health-related disciplines. A scoping review method was selected as a method to outline different types of evidence on the area of interest and to fill in the gaps for further research. The proposed review will be guided by the methodological frameworks proposed by Arksey and o’Malley and from the Joanna Briggs Institute Tricco et al. [26–29]. Thus, the following five steps will be followed in this scoping review: (i) identifying the research question, (ii) identifying relevant studies, (iii) selection of eligible studies, (iv) charting the data, and (v) collating and summarizing the results. Quality appraisal of studies will not be conducted as this review aims to explore the general scope of research conducted in this field.

### Identifying the research question

The main research question is “What are the data capturing and storage methods in forensic medicine and pathology?”

The research sub-questions are as follows:

- What data recording and reporting methods are used in forensic medicine and pathology internationally?
- What are the data recording and reporting methods used in forensic medicine and pathology locally (in the South African context?)
- What are the documented and/or reported impact of improved data recording and reporting on training, research, and the ability to provide national/provincial statistics?
- What is the value of data management systems in medicine and in health reporting or other uses/benefits reported?

**Fig. 1:**
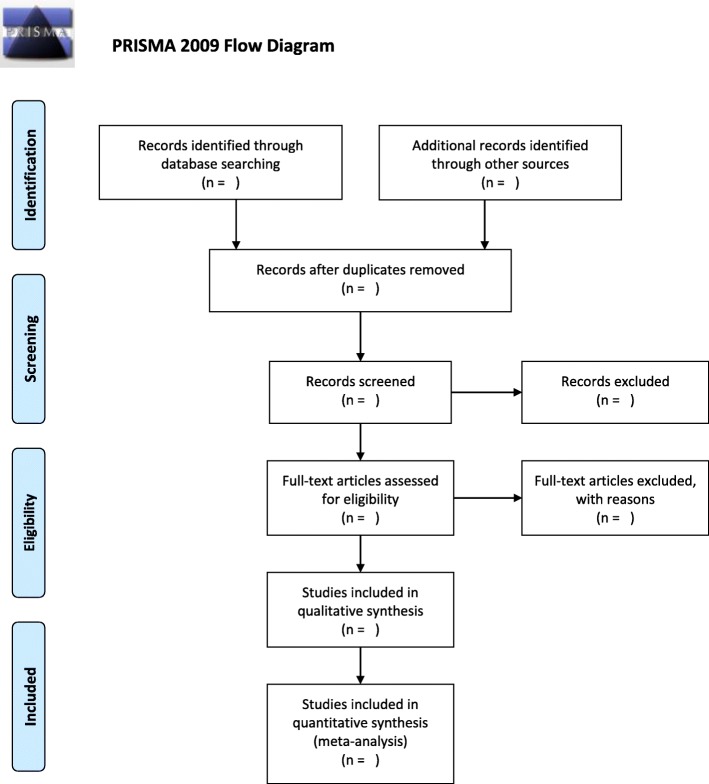
PRISMA 2009 flow diagram. From [30] Moher D et.al

This study will use the PRISMA framework (Fig. 1) to align study selection with the research question and will follow the relevant aspects of the Preferred Reporting Items for Systematics Review and Meta-Analysis Protocols (PRISMA-P) extension for scoping reviews guidelines (Table 1) to ensure thorough reporting and mapping of the body of literature [31].

**Table 1:**
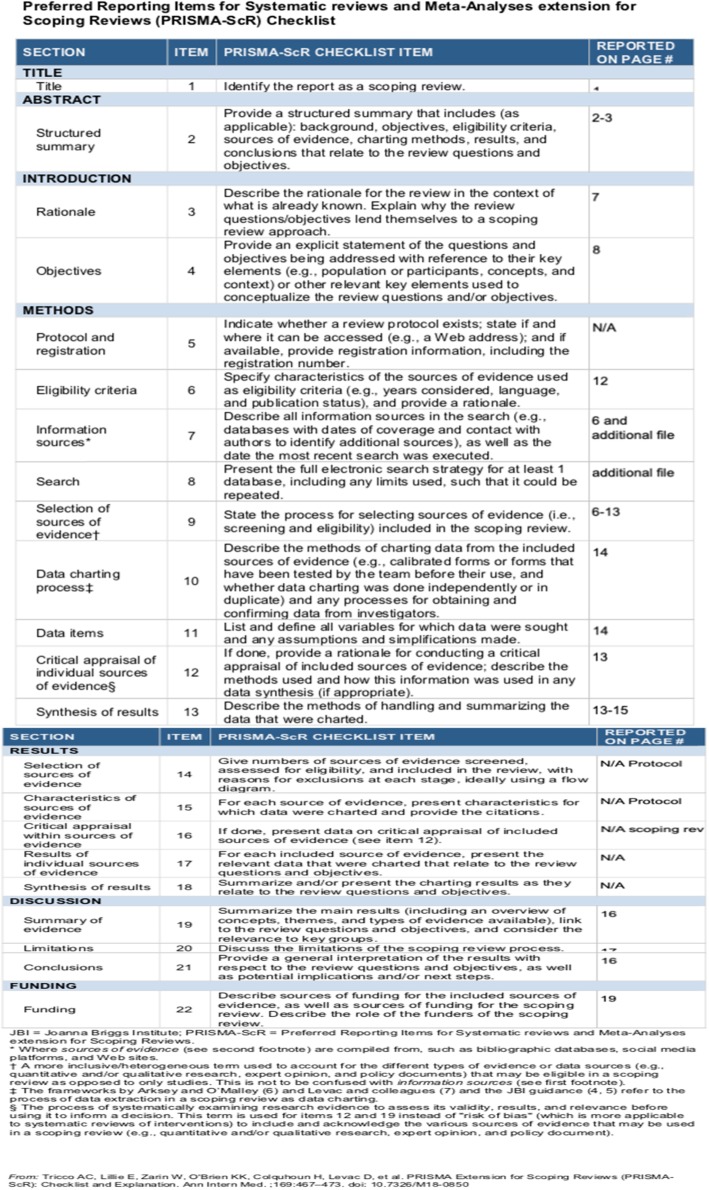
Preferred Reporting Items for Systematics Review and Meta-Analysis Protocols (PRISMA-P) extension for scoping reviews guidelines

### Identifying relevant studies

A search will be conducted for published and gray literature on the research area from January 2008. The search strategy has been included in a separate file as an addendum. The searches will be limited to articles in English and published from 2008. A hand or manual search will be conducted of the references of the included studies and websites such as the World Health Organization (WHO) and Department of Health (Provincial, National, and International). We will also examine reference lists for additional relevant articles (searching manually).

The results will be reviewed by the research team to ensure validity of the search strategy. The research team (primary and secondary author) will reach a percentage agreement of at least 50% on the included studies (Due to the complex and specialized area of field, the research is being conducted on the primary and secondary author will have to reach the percentage agreement for the study to be included. If not, the study will be excluded with reasons given from the team). Results from each database search, and from manual searches, will be exported to a single library in the EndNote X9 software (Thomson Reuters, 2016). Duplicate studies will be identified and removed using the EndNote program. The electronic database search will be recorded in a table (Table 2).

**Table 2:** Electronic Database Search Recording Table

Date of search	Electronic database	Keywords searched	Number of studies retrieved	Number of studies selected

### Selection of eligible studies

Title and abstract screening will be guided by the PRISMA framework (Tables 2 and 3) [31]. Application of further eligibility criteria will ensure that the content of the included studies is relevant to the aim of the study and the research question. The articles will be assessed for relevance and will be inclusive of all types of literature including review articles, commentary articles, editorials, abstracts from conferences, and other studies including empirical studies with all types of methodology (quantitative, qualitative, mixed, etc.).

**Table 3:** Data charting form

Data chart heading	Description
Author	Name of author/s
Date	Date article sourced
Title of study	Title of the article or study
Publication year	Year that the article was published
Publication type	Journal, website, conference, etc.
Study details and design (if applicable)	Type of study, empirical or review, etc.
Keywords	What key words were present
Abstract screening	Screening for appropriateness of article
Study sector/setting	Country/state/hospital/mortuary
Study population	Population studied with regard to demographics
Number of reviewers	Number of reviewers reviewing article
Types of data sources included	Detail the data sources
Reported impact or benefits	List the impact reported, intervention, comparison
Reported challenges or limitations	What challenges were encountered
Conclusion	Important aspects of the conclusion
Most significant findings	Noteworthy results of the study
Most relevant findings	Findings that contribute to the research question

#### Inclusion criteria

For studies to be included, they must meet the following criteria:

- Articles on medical data capturing
- Articles on data capturing of unnatural deaths, autopsies/postmortem examinations, or forensic medicine and pathology
- Conducted internationally or nationally
- Report on current data selection/management practices and the impact of the systems
- Published from 1 January 2008 until current
- Primary qualitative and quantitative studies and abstracts from conferences will be included.
- Available in English

#### Exclusion criteria

Studies will be excluded if they have any of the following characteristics:

- Data collection and management practices not relating to medicine
- Studies focusing only on case reporting
- Studies where full text article could not be obtained

The two reviewers (primary and secondary authors) will use the inclusion criteria to determine eligibility of the selected and identified studies for the review and will subsequently conduct article and full-text screening of all eligible articles. Articles will be selected on a minimum agreement of at least 50% between the two reviewers due to the complex and specialized field the review will entail (data management in forensic medicine/medicine related to autopsy reporting). An agreement will be reached after discussion until a consensus is reached. Bibliographic details, study design, intervention, comparison, study setting, funding sources, and conclusions are some of the data information that will be extracted. All attempts will be made to obtain full-text copies of selected articles, by engaging with the University of Kwazulu Natal librarian or contacting the author if necessary via email and via the University of Kwazulu Natal library.

The selection process will follow the recommendations in the Preferred Reporting Items for Systematic Reviews and Meta-Analyses Extension for Scoping Reviews (PRISMA-ScR) checklist [25] and be mapped using the PRISMA-P chart and the percentage agreement between the two reviewers.

#### Charting the data

A data charting form will be used to electronically capture relevant information from each included study. The extracted data will include the following fields (Table 3).

### Collating, summarizing, and reporting the results

A narrative report will be produced to summarize the extracted data around the following outcomes: efficiency of data capturing and database systems; utility of data systems; and impact on research, statistics, and training. These results will be described in relation to the research question and in the context of the overall study purpose. Gap identification will detect areas, such as countries that lack research on proper data capturing practices and paucity of data on data capturing.

## Discussion

This scoping review aims to identify and describe the databases use and value in the department of forensic medicine and the benefits of its use to be available for department development, epidemiological research, statistics, health trends, social development public interventions, and research.

Electronic data capturing has the potential to greatly reduce the time and costs associated with data collection. Internationally, primary health care databases are used to provide anonymized electronic health data that is utilized for research that serve both observational and interventional purposes [32, 33]. The development of software and tools to improve recording systems to support practitioners and other groups involved in health care advancement has been on a steady rise [34, 35]. In the Western Cape of South Africa Forensic Pathology Services, an excel spread system is utilized to save data and information from autopsies. In 1975, the College of American Pathologists developed a computerized National Autopsy Databank to serve as a source of pathological, biomedical, demographic, and epidemiological information to benefit research [31, 32]. It has shown how this has greatly increased research output. In the current technological age, this is now possible and realistic to create an accessible repository of records stored in an electronic format.

The database developed by Drs. Moore, Berman, and Hutchins consists of over 49,000 autopsy fact sheets contributed by numerous academic medical institutions. To date, 1200 research papers have been published using the records from that database. An autopsy database that contains information of value for epidemiologists and other researchers is technically feasible using current technology and can be designed to protect patient privacy [22, 23, 36, 37]. Placing the autopsy database on the computer maximizes its access to researchers interested in using or contributing to the database also thus improving standards [24, 25]. This review is the first part of a study to develop guidelines for efficient data capturing practices in forensic medicine which has not been reported on previously according to the authors’ knowledge. The results of this review will provide an understanding of the international and other provincial databases and their utility which will assist in developing new methods of data management in medicine. This review also has the potential to create greater awareness of how postmortem information can be used to develop and promote scientific studies to establish new medical information for use in teaching, statistics reporting, and trauma and injury progression process to improve patient care and for promotion of public health and safety. It is an important topic to support the transition to efficient reporting in the forensic medicine department and is envisioned to provide a platform that can be used in other medical departments.

## Limitations

Currently, only data reporting and management practices used in forensic medicine, in medicine, or in autopsies will be used. This may exclude studies in other departments.The keywords to be used in the search strategy are broad and may not identify specialized studies in data management. Only articles in English will be used.

## Data Availability

All data generated or analyzed during this study will be included in the published scoping review article. This will include all search results, list of excluded studies, spreadsheets, and data used for the meta-analyses, and reasons for why the selected studies were included will also be published in the article. This will ensure transparency and reproducibility.
